# Supplementary material for: Transcription Factor ERF194 Modulates the Stress-Related Physiology to Enhance Drought Tolerance of Poplar
Source: Int J Mol Sci. 2023 Jan 2;24(1):788. doi: 10.3390/ijms24010788 (PMC9821289; doi:10.3390/ijms24010788)
Supplement: Supplementary file 1 [file ijms-24-00788-s001.zip › Additional file S1.pdf]

Table S1. Detection of cis-acting elements in the promoters of genes using PlantCRAE.

**Cis-elements in Promoter Regions**											
Element		***Promoter***									
Common Name	***Function Annotation***	***Potri.001G070900***	***Potri.001G084000***	***Potri.002G013200***	***Potri.008G065600***	***Potri.018G063300***	***Potri.005G195600***	***POD2 (Potri.016G132700)***	***POD3 (Potri.007G053400)***	***SOD2 (Potri.009G005100)***	***ERF194***
TGACG-motif	cis-acting regulatory element involved in the MeJA-responsiveness		2	2	2	1	1		1		3
TCA-element	cis-acting element involved in salicylic acid responsiveness		2					1			3
TC-rich repeats	cis-acting element involved in defense and stress responsiveness	2	1				1	2			1
MRE	MYB binding site involved in light responsiveness			2		1	1			3	1
G-box	cis-acting regulatory element involved in light responsiveness		1	6	2		3		6		6
CGTCA-motif	cis-acting regulatory element involved in the MeJA-responsiveness		2	2	2	1	1		1		3
Sp1	light responsive element			1							1
AE-box	part of a module for light response					1			1		2
GC-motif	enhancer-like element involved in anoxic specific inducibility				1					1	1
MBS	MYB binding site involved in drought-inducibility				1	1	1		2		1

3-AF1 binding site	light responsive element										1
O2-site	cis-acting regulatory element involved in zein metabolism regulation				2						3
CAT-box	cis-acting regulatory element related to meristem expression								1		1
ABRE	cis-acting element involved in the abscisic acid responsiveness	1	3	5	2		3		8		7
G-Box	cis-acting regulatory element involved in light responsiveness	1	1	1			1		2		4
Box 4	part of a conserved DNA module involved in light responsiveness	5	6	6	4	7	10	6	6	5	9
Box II	part of a light responsive element				1		2				1
ARE	cis-acting regulatory element essential for the anaerobic induction	2	1	1	2	3			2	2	3
GATA-motif	part of a light responsive element	1					1	1	1		
AT1-motif	part of a light responsive module	1	1	1		2	2			1	
LTR	cis-acting element involved in low-temperature responsiveness	1	4						1		
TGA-element	auxin-responsive element	1			2						
GT1-motif	light responsive element	3	1	2		5	1	1		2	
TCT-motif	part of a light responsive element	3	1	1	4		1			1	

ATCT-motif	part of a conserved DNA module involved in light responsiveness	1		1			1		1		
P-box	gibberellin-responsive element		3						1		
circadian	cis-acting regulatory element involved in circadian control		1	1	2			1	1		
chs-CMA2a	part of a light responsive element		1								
CCAAT-box	MYBHv1 binding site			1			2				
A-box	cis-acting regulatory element			1	2						
LS7	part of a light responsive element			1							
TATC-box	cis-acting element involved in gibberellin-responsiveness						1	1	1		
AT-rich element	binding site of AT-rich DNA binding protein (ATBP-1)						1			1	
GCN4_motif	cis-regulatory element involved in endosperm expression							1	1		
LAMP-element	part of a light responsive element				1						
TCCC-motif	part of a light responsive element				1	1					
I-box	part of a light responsive element									1	
AT-rich sequence	element for maximal elicitor-mediated activation (2copies)							1			
GARE-motif	gibberellin-responsive element							2			

ACA-motif	part of gapA in (gapA-CMA1) involved with light responsiveness					1					
ACE	cis-acting element involved in light responsiveness					1					

Note:The cis-acting element *ERF19*4 could bind to were marked in RED
